# Effects of physical activity dose on cognitive ability in Chinese adolescent students: parallel mediating roles of sleep quality and smartphone addiction

**DOI:** 10.3389/fpsyg.2025.1680936

**Published:** 2025-10-31

**Authors:** Keqiang Li, Qianjin Wang, Jianye Li

**Affiliations:** ^1^Faculty of Sports Science, Wenzhou Medical University, Wenzhou, China; ^2^Department of Physical Education, Shanxi Medical University, Jinzhong, China

**Keywords:** physical activity, cognitive failures, sleep quality, smartphone addiction, adolescents

## Abstract

**Introduction:**

Adolescence is a critical developmental period marked by heightened vulnerability to cognitive and psychological disruptions. Although physical activity has been linked to improved mental health and cognition, the underlying behavioral mechanisms remain insufficiently explored. This study investigated the associations between physical activity and cognitive failures in adolescents, with sleep quality and smartphone addiction examined as parallel mediators.

**Methods:**

A cross-sectional survey was conducted among 522 adolescents (Mean age = 15.52, SD = 1.28), who completed validated questionnaires assessing physical activity (IPAQ-SF), sleep quality (PSQI), smartphone addiction (SAS-SV), and cognitive failures (CFQ).

**Results:**

Structural equation modeling revealed that higher physical activity levels were associated with fewer cognitive failures, both directly and indirectly through better sleep quality and lower smartphone addiction. Group comparisons further indicated that adolescents with high physical activity reported significantly better sleep, reduced smartphone dependence, and fewer cognitive lapses compared to those with moderate or low activity levels. Correlational analysis supported these associations.

**Discussion:**

Moderate or low activity levels. Correlational analysis supported these associations. Although causal inferences cannot be drawn due to the study’s cross-sectional design, the findings suggest that physical activity may be linked to improved cognitive functioning through modifiable behavioral factors. These results provide a foundation for future longitudinal and intervention-based research aiming to enhance adolescent cognitive health through lifestyle-based strategies.

## 1 Introduction

Adolescence is a critical developmental stage characterized by rapid and dynamic changes across physiological, psychological, and neurological domains. These transformations collectively lay the foundation for long-term cognitive and emotional health, with far-reaching implications for educational attainment, mental well-being, and social integration (Engdawork et al., 2025; Shahzad et al., 2024). During this sensitive period, the development of cognitive control, attention regulation, memory consolidation, and executive functioning undergoes significant maturation, rendering adolescents particularly susceptible to environmental and behavioral influences that may either promote or hinder neurocognitive outcomes (Selemon, 2013). As such, identifying modifiable lifestyle factors that can support optimal cognitive development during adolescence is a pressing public health and educational priority.

Among these factors, physical activity has garnered substantial empirical support as a positive contributor to adolescent cognitive functioning. Accumulating evidence suggests that regular physical activity enhances various cognitive domains, including working memory, attentional control, processing speed, and academic performance (Donnelly et al., 2016; Mura et al., 2015). These benefits are attributed to both acute and chronic physiological mechanisms, such as increased cerebral blood flow, upregulation of neurotrophic factors (e.g., BDNF), improved insulin sensitivity, and enhanced synaptic plasticity (Colucci-D’Amato et al., 2020). However, despite the established associations, the underlying behavioral and neurocognitive mechanisms through which physical activity exerts its effects on adolescent cognition remain insufficiently elucidated, particularly within the context of pervasive digital technology use.

Contemporary adolescents inhabit an increasingly digitalized environment, with unprecedented levels of exposure to smartphones, social media, and online entertainment. While digital technologies offer cognitive stimulation and opportunities for learning, excessive smartphone use—especially in the form of compulsive or addictive behaviors—has emerged as a salient concern (Wacks and Weinstein, 2021). A growing body of research links high levels of smartphone use to negative cognitive outcomes, such as impaired attention, working memory deficits, cognitive fatigue, and reduced academic engagement (Anwar and Abbas, 2024; Liebherr et al., 2020). The cognitive overload hypothesis posits that frequent digital multitasking taxes limited attentional resources, thereby compromising higher-order executive functions and increasing susceptibility to everyday cognitive errors (Zehra and Malik, 2025; Schmitt et al., 2021). Furthermore, smartphone overuse—particularly during pre-sleep hours—has been shown to disrupt circadian rhythms by suppressing melatonin secretion, delaying sleep onset, and fragmenting sleep architecture, all of which contribute to cognitive impairments the following day (Höhn et al., 2024; Claudatos et al., 2019).

Sleep quality, in this context, emerges as a pivotal mediator of adolescent cognitive health. Sleep plays a vital restorative role, facilitating memory consolidation, emotional regulation, and metabolic homeostasis (Kufel et al., 2025). Poor sleep quality and insufficient sleep duration have been consistently linked to attentional lapses, decision-making deficits, and diminished learning capacity in adolescents (Satterfield and Killgore, 2019; Telzer et al., 2013). Neurophysiological evidence underscores sleep’s critical function in synaptic homeostasis and glymphatic clearance, suggesting that disrupted sleep may directly compromise brain function and structural integrity (van Hattem et al., 2025; Reiter et al., 2023). Importantly, physical activity has also been recognized as a non-pharmacological intervention for improving both sleep quality and circadian stability, further reinforcing its potential role in cognitive enhancement (Blackman et al., 2021).

Given these complex interrelations, it is plausible that the cognitive benefits of physical activity are not solely attributable to direct neurobiological effects, but are also mediated by modifiable behavioral pathways—most notably sleep quality and digital media use. Despite extensive literature examining each of these domains in isolation, relatively few studies have adopted an integrated framework to simultaneously investigate the mediating roles of sleep and smartphone addiction in the relationship between physical activity and cognitive functioning among adolescents. Furthermore, most extant research tends to focus narrowly on specific cognitive domains, such as executive function or working memory, which may not fully capture the broader spectrum of real-world cognitive functioning (Kalén et al., 2021).

To address these gaps, the present study adopts a more ecologically valid approach by assessing everyday cognitive failures—such as attentional lapses, memory errors, and action slips—using the well-validated Cognitive Failures Questionnaire (CFQ). This measure reflects not only latent cognitive ability but also the practical implications of cognitive dysfunction in academic and social contexts. Grounded in the behavioral pathway model of cognition, which posits that lifestyle behaviors influence cognitive outcomes via modifiable mediators such as sleep and media use patterns (Grasset et al., 2022; Lemes et al., 2021), this study proposes a parallel mediation model in which sleep quality and smartphone addiction operate as independent, concurrent mediators in the relationship between physical activity and cognitive failures.

Specifically, we hypothesize that:

(1)  Higher levels of physical activity are negatively associated with cognitive failures;
(2)  The relationship is partially mediated by improved sleep quality and reduced smartphone addiction; and.
(3)  The two mediators exert distinct and independent effects, representing complementary behavioral pathways through which physical activity influences cognitive performance.
(4)  Different levels of physical activity have significant effects on adolescents’ cognitive levels.

By integrating theoretical perspectives and empirical insights from exercise psychology, sleep science, and digital media research, this study seeks to provide a comprehensive understanding of the multifaceted mechanisms linking physical activity to cognitive development in adolescents navigating the challenges of a hyper-connected digital age. Such insights may inform targeted interventions and public health strategies aimed at fostering healthier lifestyles and enhancing cognitive resilience among youth populations.

## 2 Materials and methods

### 2.1 Participants

A total of 522 adolescents (Mage = 15.52, SD = 1.28; 48.5% female and 51.5% male) were recruited from three middle schools in Zhejiang Province, China using a cluster sampling method. Inclusion criteria were: (1) aged between 13 and 18 years; (2) attending full-time school; and (3) able to read and complete the questionnaire independently. Participants with confirmed neurological or psychiatric disorders were excluded. Informed consent was obtained from parents and assent from adolescents before participation. The study protocol was approved by the Ethics Committee of Shanxi Medical University. It was a quantitative, correlational, and descriptive cross-sectional study conducted as part of a research project and registered in the protocol registry.^1^^,^^2^ All procedures adhered to the Declaration of Helsinki.

A prior power analysis conducted via G*Power 3.1 indicated that a sample size of at least 300 participants would be required to detect a medium-sized mediation effect (*f*^2^ = 0.15) with 80% power and *α* = 0.05 in a model containing two parallel mediators. The final sample size was 553. After eliminating invalid samples, the effective sample size was 522, which exceeded this threshold and ensured that all planned analyses had sufficient statistical power.

### 2.2 Procedure

This study adopted a cross-sectional online survey design. Data were collected from October to December 2024 in a group setting during regular school hours, supervised by trained research assistants. Participants completed a battery of self-report questionnaires measuring physical activity, sleep quality, smartphone addiction, and cognitive impairment. The questionnaires took approximately 20 min to complete. To ensure data integrity, the instructions were standardized and anonymity was guaranteed. Questionnaires were checked after submission to minimize missing data. Informed consent was obtained from the students’ parents before filling out the questionnaires. The research protocol was approved by the Shanxi medical university, approve ID was SXMU2025331, and all procedures adhered to the Declaration of Helsinki and applicable national research ethics guidelines.

### 2.3 Instruments

#### 2.3.1 Physical activity

The Physical activity was measured using the International Physical Activity Questionnaire – Short Form (IPAQ-SF), developed by Craig et al. (2003). The instrument includes 7 items evaluating the frequency and duration of vigorous-intensity activity, moderate-intensity activity, walking, and sedentary behavior during the previous 7 days. Each activity is assigned a Metabolic Equivalent (MET) score: walking = 3.3 METs, moderate = 4.0 METs, vigorous = 8.0 METs. Total physical activity is calculated in MET-minutes/week using the formula: >MET level × minutes of activity × days per week. For subgroup comparisons, participants were categorized into three physical activity levels according to standard IPAQ scoring guidelines: Low (<600 MET-min/week), Moderate (600–2,999 MET-min/week), and High (≥3,000 MET-min/week). Example items include: “During the last 7 days, on how many days did you do vigorous physical activities like heavy lifting, digging, aerobics, or fast bicycling?”; “How much time did you usually spend doing moderate physical activities on one of those days?” The IPAQ-SF has been widely validated across multiple populations and shows acceptable reliability. In this study the Cronbach’s alpha was 0.903.

#### 2.3.2 Sleep quality

Sleep quality was assessed using the Pittsburgh Sleep Quality Index (PSQI), originally developed by Buysse et al. (1989). This 19-item scale evaluates seven components of sleep over the past month: subjective sleep quality, sleep latency, sleep duration, habitual sleep efficiency, sleep disturbances, use of sleeping medication, and daytime dysfunction. Each component is scored on a 0–3 scale and summed to yield a global score ranging from 0 to 21, where higher scores indicate poorer sleep quality. A global PSQI score >5 is commonly used as the cutoff for distinguishing good versus poor sleepers. Example items include: “During the past month, how long (in minutes) has it usually taken you to fall asleep each night?”; “During the past month, how often have you had trouble staying awake while driving, eating meals, or engaging in social activity?” The PSQI has demonstrated good internal consistency in adolescent samples. In this study the Cronbach’s alpha was 0.921.

#### 2.3.3 Smartphone addiction

Problematic smartphone use was evaluated using the Smartphone Addiction Scale – Short Version (SAS-SV), developed by Kwon et al. (2013). The SAS-SV consists of 10 items assessing withdrawal, daily-life disturbance, positive anticipation, tolerance, and cyberspace-oriented relationship. Each item is rated on a 6-point Likert scale (1 = strongly disagree, 6 = strongly agree), yielding a total score range from 10 to 60, with higher scores reflecting greater levels of smartphone addiction. It identifies the different ranges for males and females. Males are addicted to scores higher than 31, with a high risk of addiction with scores between 22 and 31, and females are addicted to scores higher than 33, with a high risk of addiction on scores between 22 and 33. Example items include: “I have missed planned work due to smartphone use.”; “I feel impatient and fretful when I am not holding my smartphone.”; “I have difficulty concentrating in class, while doing assignments, or while working due to smartphone use.” The SAS-SV has shown excellent internal reliability and has been validated in Chinese youth samples. In this study the Cronbach’s alpha was 0.966.

#### 2.3.4 Cognitive failures

Cognitive performance was assessed using the Cognitive Failures Questionnaire (CFQ), developed by Broadbent et al. (1982). The CFQ is a 25-item self-report scale measuring lapses in attention, action, perception, and memory over recent weeks. Responses are rated on a 5-point scale (0 = never, 4 = very often), resulting in total scores ranging from 0 to 100, with higher scores reflecting greater levels of cognitive failures. Example items include: “Do you forget why you went from one part of the house to another?”; “Do you find you confuse left and right when giving directions?”; “Do you fail to notice signposts on the road?” The CFQ has demonstrated strong psychometric properties, with Cronbach’s *α* ranging from 0.85 to 0.91 across adolescent and adult populations. The Chinese version has been validated with acceptable internal consistency. In this study the Cronbach’s alpha was 0.983.

### 2.4 Data analysis

Data were analyzed using IBM SPSS 26.0 and the PROCESS macro program (Model 4). Preliminary data screening and variable coding were performed using Microsoft Excel prior to analysis. First, descriptive statistics, reliability analyses, and Pearson correlation coefficients were calculated for all study variables. Second, normality was assessed using skewness, kurtosis, the Shapiro–Wilk test, and the Kolmogorov–Smirnov test. Because parametric methods are robust in large samples (*N* = 300), variables that did not meet strict normality assumptions were retained. Third, factor loadings were calculated for all variables; variables below 0.5 were considered underrepresented and removed. Model fit coefficients were also calculated, using *χ*^2^/df, CFI, TLI, RMSEA, and SRMR as reference goodness-of-fit indicators. In the fourth step, parallel mediation analyses were conducted to examine whether sleep quality (PSQI) and smartphone addiction (SAS-SV) independently mediated the relationship between physical activity (IPAQ-SF) and cognitive impairment (CFQ). Confidence intervals for the indirect effect were estimated using bootstrapping, and bias-corrected 95% confidence intervals (CIs) for the indirect effect were calculated using a bootstrap procedure with 5,000 resamples. Indirect effects were considered statistically significant if their CIs did not include zero.

## 3 Results

### 3.1 Sample description

Table 1 shows the final sample consisted of 522 adolescents, with a mean age of 15.52 years (SD = 1.28), ranging from 13 to 18 years. The gender distribution was relatively balanced, with 253 females (48.5%) and 269 males (51.5%). Participants were categorized into physical activity levels according to the IPAQ-SF scoring guidelines. Specifically, 72 participants (13.8%) were classified as engaging in high levels of physical activity, 362 participants (69.3%) met the criteria for moderate activity, and 88 participants (16.9%) were categorized as having low activity levels. These results suggest that the majority of the sample reported at least moderate physical activity, providing a suitable basis for subsequent analysis of behavioral and cognitive outcomes.

**Table 1:** Sample characteristics (*n* = 522).

Characteristic	Total sample
Age [Mean (SD)]	15.52 (1.28)
Gender	
Female	253
Male	269
Physical activity level	
High	72
Moderate	362
Low	88

**Figure 1:**
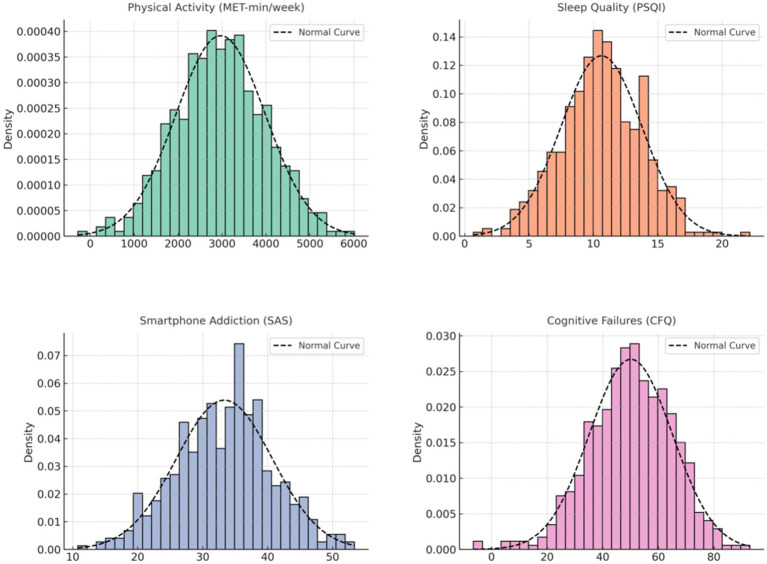
Normal distribution plot.

Descriptive statistics and normality test results for all continuous variables are presented in Table 2 and Figure 1. The mean level of physical activity was 3006.11 MET-min/week (SD = 1016.86), indicating a generally active sample. The average sleep quality score (PSQI) was 10.76 (SD = 3.19), suggesting that many participants experienced suboptimal sleep quality. The mean smartphone addiction score was 32.98 (SD = 7.97), while the average cognitive failures score was 50.28 (SD = 14.95), reflecting moderate levels of self-reported cognitive lapses. Skewness (S) and kurtosis (K) values for all variables ranged from −0.496 to 0.248, falling within acceptable thresholds (±1), which suggests approximate normality in distribution. However, results from the Kolmogorov–Smirnov (K–S) and Shapiro–Wilk (S–W) tests varied by variable.

**Table 2:** Descriptive statistics (*n* = 522).

Variables	Mean	SD	*S*	*K*	Kolmogorov–Smirnov		Shapiro–Wilk	
					*D*	*P*	*W*	*P*
Physical activity	3006.114	1016.862	0.139	−0.147	0.034	0.180	0.997	0.358
Sleep quality	10.764	3.187	0.103	−0.496	0.157	0.000**	0.925	0.000**
Smartphone addiction	32.982	7.974	0.172	0.012	0.063	0.000**	0.995	0.090
Cognitive failures	50.280	14.951	0.053	0.248	0.075	0.000**	0.988	0.000**

**p < 0.01.

For physical activity, both K–S (*p* = 0.180) and S–W (*p* = 0.358) tests indicated non-significant results, supporting a normal distribution.

In contrast, sleep quality significantly deviated from normality in both tests (K–S *p* < 0.001, S–W *p* < 0.001). For smartphone addiction, the S–W test was non-significant (*p* = 0.090), but the K–S test showed marginal significance (*p* < 0.001). Cognitive failures also demonstrated significant deviation from normality based on the S–W test (*p* < 0.001), though skewness and kurtosis remained within normal range.

Given the large sample size (*n* > 500), these slight violations of normality are not unexpected. Furthermore, the skewness and kurtosis values indicate that the deviations are unlikely to meaningfully affect the validity of parametric analyses such as regression or mediation models (Blanca et al., 2013).

### 3.2 Variables correlation analysis

The Bivariate Pearson correlation coefficients among the study variables are presented in Table 3. As expected, physical activity was significantly and negatively correlated with sleep quality (*r* = −0.707, *p* < 0.001) and cognitive failures (*r* = −0.584, *p* < 0.001), indicating that individuals with higher levels of physical activity reported better sleep and fewer cognitive lapses.

**Table 3:** Correlation analysis.

Variables	Physical activity	Sleep quality	Smartphone addiction	Cognitive failures
Physical activity	1			
Sleep quality	−0.707**	1		
Smartphone addiction	−0.273**	0.210**	1	
Cognitive failures	−0.584**	0.594**	0.443**	1

**p < 0.001.

Physical activity was negatively associated with smartphone addiction (*r* = −0.273, *p* < 0.001), suggesting that more physically active adolescents tend to exhibit fewer problematic smartphone use behaviors.

Conversely, sleep quality was positively correlated with cognitive failures (*r* = 0.594, *p* < 0.001), indicating that poorer sleep is associated with more frequent cognitive lapses. Furthermore, smartphone addiction showed a significant negative correlation with cognitive failures (*r* = −0.443, *p* < 0.001), suggesting that higher levels of smartphone use problems may be linked to lower cognitive performance.

All correlations were statistically significant at the *p* < 0.001 level, and the direction and magnitude of the associations were consistent with theoretical expectations and previous research findings. These results provide preliminary support for the hypothesized mediation model.

### 3.3 Parallel mediation model analysis

The goodness-of-fit indices for the measurement model are presented in Table 4. Overall, the model demonstrated an excellent fit to the data across multiple evaluation criteria. The chi-square to degrees of freedom ratio (*χ*^2^/df) was 1.042, well below the recommended threshold of 3.00.

**Table 4:** Model fit indices for the measurement model.

Fit index	Value	Acceptable threshold
*χ*^2^/df	1.042	<3.00
Comparative Fit Index (CFI)	0.998	≥0.90
Tucker–Lewis Index (TLI)	0.998	≥0.90
Root Mean Square Error of Approximation (RMSEA)	0.009	≤0.10
Standardized Root Mean Square Residual (SRMR)	0.021	≤0.10

The Comparative Fit Index (CFI) and the Tucker–Lewis Index (TLI) were both 0.998, exceeding the conventional cutoff value of 0.90, suggesting strong comparative and incremental fit relative to the null model. Furthermore, the Root Mean Square Error of Approximation (RMSEA) was 0.009, and the Standardized Root Mean Square Residual (SRMR) was 0.021, both of which are below the commonly accepted threshold of 0.10, indicating low residual error and excellent approximation in the population (Mulaik et al., 1989).

Collectively, these fit indices provide strong support for the construct validity and factorial structure of the latent variables included in the study (i.e., physical activity, sleep quality, smartphone addiction, and cognitive failures). The results indicate that the measurement model adequately represents the observed data and is suitable for further structural equation modeling.

**Figure 2:**
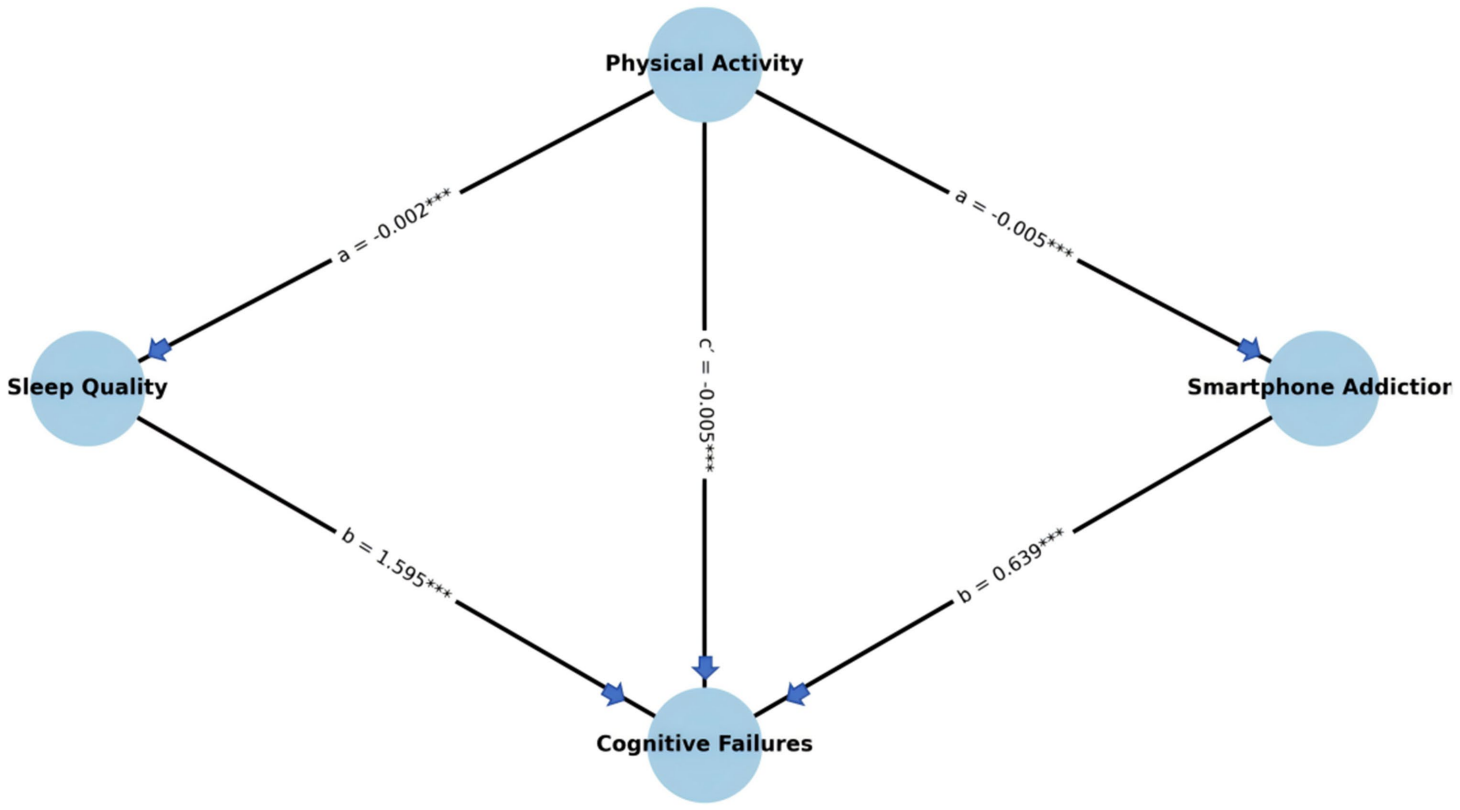
Parallel mediation model path diagram. Values represent the significant standardized regression coefficients. **p < 0.01.

A parallel mediation analysis was conducted to examine whether sleep quality and smartphone addiction mediated the relationship between physical activity and cognitive failures, using a bias-corrected bootstrapping procedure with 5,000 resamples. The results are detailed below and summarized in Table 5 and Figure 2.

**Table 5:** Bootstrap analysis of the mediation effect size and significance test.

Path			Standardized Effect Size (Effect)	95% CI		*p*
				LL	UL	
IPAQ= > PSQI= > CFQ	a*b	Indirect effect	**−0.003**	−0.270	−0.172	0.000
IPAQ= > PSQI	a	X= > M	**−0.002**	−0.002	−0.002	0.000
PSQI= > CFQ	b	M= > Y	**1.595**	1.295	1.895	0.000
IPAQ= > CFQ	c’	Direct effect	**−0.005**	−0.006	−0.004	0.000
IPAQ= > CFQ	c	Total effect	**−0.011**	−0.012	−0.010	0.000
IPAQ= > SAS= > CFQ	a*b	Indirect effect	**−0.003**	−0.238	−0.160	0.000
IPAQ= > SAS	a	X= > M	**−0.005**	−0.005	−0.004	0.000
SAS= > CFQ	b	M= > Y	**0.639**	0.527	0.751	0.000
IPAQ= > CFQ	c’	Direct effect	**−0.005**	−0.006	−0.004	0.000
IPAQ= > CFQ	c	Total effect	**−0.011**	−0.012	−0.010	0.000

CI, confidence interval; LL, lower limit; UL, upper limit. X independent variable, M mediating variable, Y dependent variable. **p < 0.01. Bolded values represent significant differences.

#### 3.3.1 Mediation via sleep quality

Physical activity was a significant positive predictor of sleep quality (path a: *β* = −0.002, 95% CI [−0.002, −0.002], *p* < 0.001), indicating that greater physical activity was associated with improved sleep quality. In turn, sleep quality significantly predicted cognitive failures (path b: *β* = 1.595, 95% CI [1.295, 1.895], *p* < 0.001), such that poorer sleep quality was associated with more cognitive failures. The indirect effect of physical activity on cognitive failures through sleep quality was statistically significant (*β* = −0.003, 95% CI [−0.270, −0.172], *p* < 0.001), suggesting that sleep quality partially mediated the effect of physical activity on cognitive failures.

#### 3.3.2 Mediation via smartphone addiction

Similarly, physical activity significantly predicted lower levels of smartphone addiction (path a: *β* = −0.005, 95% CI [−0.005, −0.004], *p* < 0.001), and higher smartphone addiction was positively associated with increased cognitive failures (path b: *β* = 0.639, 95% CI [0.527, 0.751], *p* < 0.001). The indirect effect of physical activity on cognitive failures through smartphone addiction was also significant (*β* = −0.003, 95% CI [−0.238, −0.160], *p* < 0.001), indicating a second significant mediation pathway.

#### 3.3.3 Direct and total effects

Even after accounting for both mediators, the direct effect of physical activity on cognitive failures remained statistically significant (*c*′ = −0.005, 95% CI [−0.006, −0.004], *p* < 0.001), demonstrating that the mediators only partially explained the association. The total effect was also significant (*c* = −0.011, 95% CI [−0.012, −0.010], *p* < 0.001), supporting a model of partial mediation.

Collectively, these findings provide robust evidence for a dual-path partial mediation model, wherein the beneficial effects of physical activity on cognitive failures are partially explained by enhanced sleep quality and reduced smartphone addiction. These results highlight two key behavioral mechanisms linking physical activity to cognitive functioning, suggesting that interventions aimed at promoting adolescent cognitive health should consider targeting both sleep hygiene and digital behavior patterns.

### 3.4 ANOVA of variables in different physical activity groups

**Figure 3:**
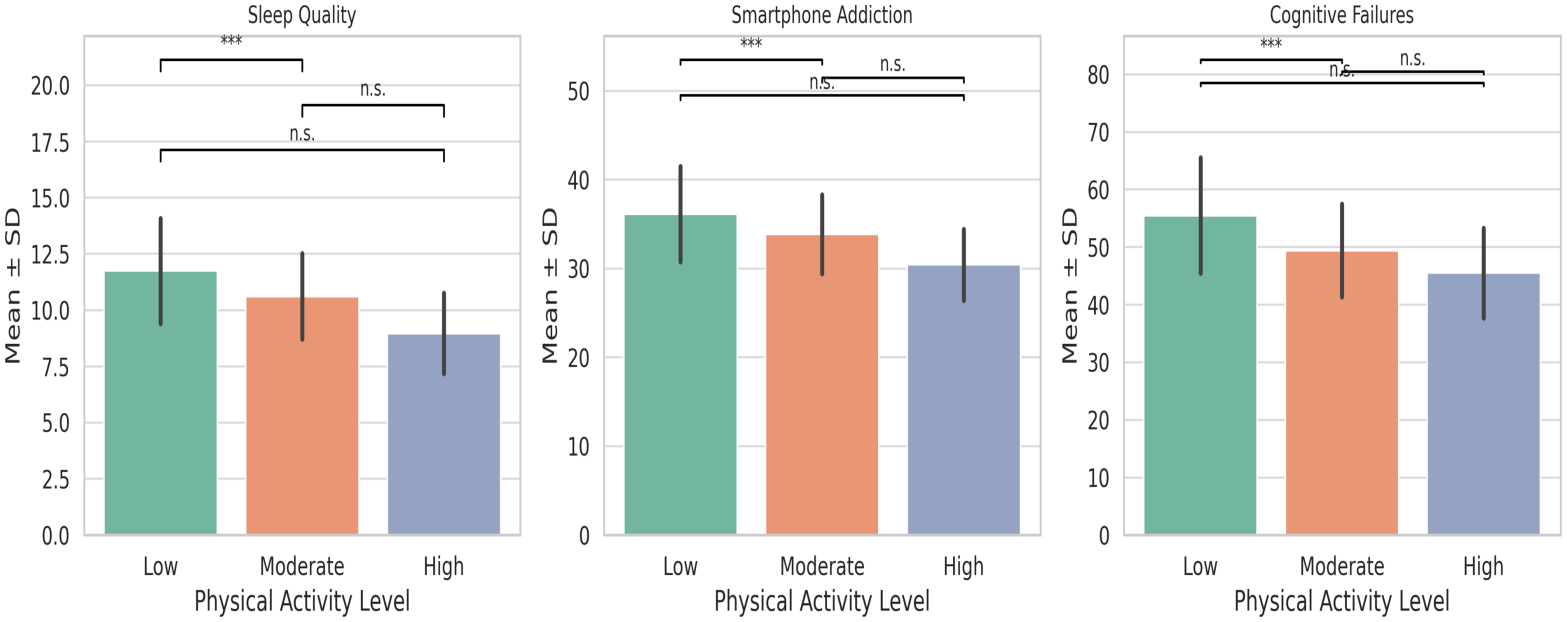
ANOVA bar chart of different physical activity groups. n.s. indicates no significant difference, *** indicates significant difference.

To examine whether psychological and cognitive outcomes differed by physical activity levels, a one-way analysis of variance (ANOVA) was conducted across three groups categorized by the International Physical Activity Questionnaire (IPAQ): low, moderate, and high physical activity. As shown in Table 6 and Figure 3, there were statistically significant group differences in all three outcome variables.

**Table 6:** Analysis of variance for different physical activity level groups.

Variables	PA_Level (mean ± SD)			*F*	*P*
	High (*n* = 88)	Low (*n* = 72)	Moderate (*n* = 362)		
Sleep quality	8.97 ± 1.78	11.75 ± 2.34	10.61 ± 1.89	43.891	0.000**
Smartphone addiction	30.43 ± 3.98	36.13 ± 5.38	33.89 ± 4.41	34.628	0.000**
Cognitive failures	45.51 ± 7.74	55.52 ± 10.05	49.43 ± 8.03	32.139	0.000**

**p < 0.01.

The ANOVA revealed a significant effect of physical activity level on sleep quality [*F*(2, 519) = 43.891, *p* < 0.001]. Participants in the high physical activity group reported better sleep quality (Mean ± SD = 8.97 ± 1.78) compared to those in the moderate (10.61 ± 1.89) and low (11.75 ± 2.34) activity groups.

A significant group effect was also observed for smartphone addiction [*F*(2, 519) = 34.628, *p* < 0.001], with individuals in the high activity group scoring significantly lower (30.43 ± 3.98) than those in the moderate (33.89 ± 4.41) and low (36.13 ± 5.38) activity groups, suggesting that increased physical activity is associated with reduced problematic smartphone use.

Similarly, significant differences were found for cognitive failures [*F*(2, 519) = 32.139, *p* < 0.001], with the high activity group reporting the fewest cognitive failures (45.51 ± 7.74), followed by the moderate (49.43 ± 8.03) and low (55.52 ± 10.05) groups.

These findings suggest that higher levels of physical activity are consistently associated with better psychological functioning and cognitive outcomes in adolescents. The results support the role of physical activity not only in physical health, but also in reducing psychological maladjustment and cognitive lapses.

## 4 Discussion

The present study aimed to investigate the mechanisms underlying the relationship between physical activity and cognitive function in adolescents, with particular emphasis on the potential parallel mediating roles of sleep quality and smartphone addiction. Our findings offer novel contributions to the literature by elucidating how lifestyle behaviors in the digital age, specifically physical activity and digital media use, jointly influence cognitive functioning in youth. By integrating these key factors, this study provides a more comprehensive understanding of the behavioral mechanisms that shape adolescent cognition.

### 4.1 Physical activity, sleep quality, and smartphone addiction: correlational findings and cognitive implications

First and foremost, consistent with a growing body of literature, our results demonstrated that higher levels of physical activity were significantly associated with better cognitive functioning, as assessed by the Cognitive Failures Questionnaire (CFQ). These findings corroborate prior research suggesting that regular physical activity enhances cognitive performance, including working memory, attention, and processing speed (De Greeff et al., 2018; Rathore and Lom, 2017). The physiological mechanisms underlying this relationship are well-documented and include increased cerebral blood flow, improved neurogenesis, and enhanced neuroplasticity. Physical activity is also known to reduce the adverse effects of stress and anxiety, which may further support cognitive efficiency by enhancing emotional regulation and mitigating the negative impact of psychological stress on attentional processes. Moreover, the positive relationship between physical activity and cognitive performance aligns with theories that emphasize the role of physical health behaviors in optimizing brain function, particularly during the critical developmental window of adolescence (Erickson et al., 2019).

Correlational analyses further substantiated these associations, demonstrating that physical activity was positively correlated with sleep quality and negatively correlated with both smartphone addiction and cognitive failures. These results underscore the multifaceted benefits of physical activity, not only in promoting physiological regulation—such as improving sleep quality—but also in mitigating maladaptive digital behaviors. The strong negative correlation observed between smartphone addiction and cognitive failures reinforces existing literature that links excessive smartphone use with impaired attention, cognitive fatigue, and poorer memory performance (Wilmer et al., 2017; Hadlington, 2015). This suggests that problematic smartphone use may deplete cognitive resources, leading to higher incidences of everyday cognitive failures, which are particularly detrimental in the academic and social realms of adolescent life. Similarly, the robust positive correlation between sleep quality and cognitive performance supports neurocognitive models that emphasize sleep’s restorative role in executive functioning and attention regulation, underscoring the importance of good sleep hygiene for optimal brain health (Malhi et al., 2015).

### 4.2 Mediating roles of sleep quality and smartphone addiction in the relationship between physical activity and cognitive outcomes

A central contribution of this study is the identification of two significant mediators in the relationship between physical activity and cognitive outcomes: sleep quality and smartphone addiction. Adolescents who engaged in higher levels of physical activity reported significantly better sleep quality, which, in turn, was associated with fewer cognitive failures. This finding is consistent with prior research showing that physical activity improves sleep architecture, increases sleep duration, and enhances sleep quality—factors critical for memory consolidation, learning, and attentional control (Sewell et al., 2021; Sejbuk et al., 2022). The standardized indirect effect through sleep quality was significant, further validating sleep as an important mediator in the relationship between physical activity and cognitive performance. This highlights the critical role of sleep in modulating the cognitive benefits of physical activity, suggesting that interventions aimed at improving sleep quality may amplify the cognitive advantages of regular exercise.

Simultaneously, our results revealed that higher levels of physical activity were associated with lower smartphone addiction scores, which in turn significantly predicted fewer cognitive failures. This novel pathway underscores the protective role of physical activity against excessive digital device use, potentially by occupying discretionary time that might otherwise be spent on smartphones, or by providing alternative avenues for psychosocial engagement and emotional regulation. The negative impact of smartphone addiction on cognitive function is well-documented, with excessive use associated with impaired executive function, attentional bias, and diminished working memory (De Barros, 2024). Our findings suggest that physical activity may help mitigate these negative effects, thereby reducing cognitive failures. This reinforces the idea that promoting physical activity may serve as an effective strategy for combating digital overuse in the adolescent population.

Furthermore, the mediation model revealed that both sleep quality and smartphone addiction partially mediated the effect of physical activity on cognitive failures, as the direct effect of physical activity remained significant. This suggests that while improvements in sleep and reductions in smartphone addiction account for part of the cognitive benefits of physical activity, additional mechanisms may also contribute. These could include psychosocial engagement, mood regulation, or neurobiological adaptations that enhance cognitive resilience in the face of digital distractions (Shalaby, 2024; Athira and Nithyanandan, 2025). The persistent direct effect of physical activity on cognitive outcomes implies that physical activity may exert a broad range of benefits, some of which may not be fully captured by the mediating factors examined in this study. Although the indirect effects of physical activity on cognitive failures through both sleep quality (*β* = −0.003) and smartphone addiction (*β* = −0.003) are statistically significant, the effect sizes are relatively small. This raises important questions about the practical implications of these findings. While these small effect sizes suggest that physical activity does contribute to a reduction in cognitive failures through these two pathways, it is essential to acknowledge that other factors may also play a significant role in influencing cognitive functioning. The small magnitude of the mediation effects does not diminish the importance of these mechanisms but rather highlights the complex and multifactorial nature of cognitive health. The small but statistically significant indirect effects observed in this study may vary depending on individual differences such as age, gender, or baseline levels of physical activity. For instance, it is possible that the mediation effects could be stronger for specific age groups or genders. Previous research has shown that physical activity has varying effects on sleep and cognitive function across different age groups (e.g., adolescents versus older adults), and it may be particularly important for younger individuals who are in critical stages of cognitive development (Kato et al., 2018).

### 4.3 One-way ANOVA of different physical activity subgroups: effects on sleep quality, smartphone addiction, and cognitive function

The analysis of variance further substantiated these mediation findings. Adolescents with high physical activity levels exhibited significantly better sleep quality, lower smartphone addiction, and fewer cognitive failures compared to those with moderate or low physical activity. These results validate the directional assumptions of the mediation model, reinforcing the notion that physical activity plays a critical role in promoting adolescent cognitive and psychological well-being. The fact that physical activity was consistently associated with improvements across multiple behavioral and cognitive domains highlights its importance as a modifiable factor in promoting adolescent health. Given the rapid digitalization of adolescents’ lives, these findings suggest that physical activity may serve as a protective buffer against the cognitive risks associated with excessive screen time.

### 4.4 Limitation

First, the cross-sectional design of the study limits the ability to establish causal relationships between physical activity, sleep quality, smartphone addiction, and cognitive outcomes. Future longitudinal studies are needed to better understand the temporal dynamics and causal pathways underlying these associations. Second, the reliance on self-report measures introduces the potential for reporting bias, particularly regarding physical activity levels and smartphone use. Objective measures of activity and screen time, such as accelerometry and smartphone tracking applications, would provide more accurate assessments of these behaviors. Finally, while the study focused on cognitive failures as a measure of cognitive performance, future research should also examine other cognitive outcomes, such as academic achievement and cognitive flexibility, to provide a more comprehensive picture of the cognitive benefits of physical activity.

In conclusion, this study provides novel insights into the mechanisms through which physical activity influences cognitive function in adolescents. By identifying sleep quality and smartphone addiction as significant mediators, our findings underscore the importance of promoting physical activity as part of a broader strategy to optimize adolescent cognitive health in the digital era. Future research should continue to explore these mechanisms and examine how interventions targeting physical activity, sleep, and digital behaviors can enhance cognitive outcomes and overall well-being in youth.

## 5 Conclusion

This study highlights a significant association between higher levels of physical activity and improved cognitive function in adolescents, with sleep quality and smartphone addiction acting as parallel mediating variables. The results suggest that physical activity contributes to reduced cognitive impairment, in part by improving sleep quality and reducing smartphone addiction, with each mediating variable independently influencing cognitive outcomes. Group comparisons based on physical activity levels further supported these relationships, showing that more active adolescents had better sleep quality, lower levels of smartphone addiction, and fewer cognitive errors. While the study’s cross-sectional design limits causal inferences, the results underscore the importance of physical activity in adolescent development and suggest that sleep quality and smartphone use are key modifiable factors for future research.

## Data Availability

The original contributions presented in the study are included in the article/supplementary material, further inquiries can be directed to the corresponding author.

## Footnotes

^1^ ClinicalTrials.gov

^2^ https://clinicaltrials.gov/ct2/show/NCT04432038
